# Inkjet‐Printed Cu_2_ZnSn(S, Se)_4_ Solar Cells

**DOI:** 10.1002/advs.201500028

**Published:** 2015-05-05

**Authors:** Xianzhong Lin, Jaison Kavalakkatt, Martha Ch. Lux‐Steiner, Ahmed Ennaoui

**Affiliations:** ^1^ Institute for Heterogeneous Material SystemsHelmholtz‐Zentrum Berlin für Materialien und EnergieHahn‐Meitner‐Platz 114109BerlinGermany; ^2^ Freie Universität BerlinBerlinGermany; ^3^ Qatar Environment and Energy Research Institute (QEERI)P.O. Box5825DohaQatar

**Keywords:** CZTSSe, inkjet printing, kesterite, solar cells

## Abstract

**Cu_2_ZnSn(S, Se)_4_‐based solar cells with total area (0.5 cm^2^) power conversion efficiency of 6.4%** are demonstrated from thin film absorbers processed by inkjet printing technology of Cu‐Zn‐Sn‐S precursor ink followed by selenization. The device performance is limited by the low fill factor, which is due to the high series resistance.

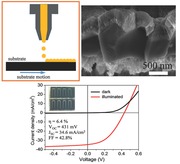

Cu_2_ZnSn(S,Se)_4_ (CZTSSe), generally called kesterite, is a p‐type semiconductor material, which can be obtained by substituting In/Ga in Cu(In,Ga)(S,Se)_2_ (CIGSSe) with Zn and Sn. Although the record efficiency of CZTSSe‐based solar cells (12.7%) is far below that of CIGSSe‐based solar cells (21.7%), CZTSSe is still quite attractive due to its abundant constituents.^1^, ^2^ Different approaches have been applied to deposit CZTSSe layers. Several techniques have been demonstrated to be effective for the deposition of CZTSSe layers such as evaporation^3^ and electrochemical deposition.^4^ Recently, very promising methods based on ink precursor routes are developed for the formation of high quality CZTSSe absorbers. These include the use of molecular precursor inks^5^, ^6^, ^7^, ^8^, ^9^ as well as monodispersed nanoparticle inks.^10^, ^11^, ^12^ The most successful method so far is to use hydrazine slurry‐based approach which gives the best device with efficiency up to 12.7%.^1^ However, hydrazine is very toxic and explosive, which is unfavorable for the implementation in large scale production. Several groups have reported on alternative solvents such as dimethyl sulfoxide (DMSO) instead of hydrazine for the formulation of Cu‐Zn‐Sn‐S inks.^5^, ^9^, ^13^ The formation of the CZTSSe thin film absorbers is achieved by annealing the spin‐coated or doctor‐bladed Cu‐Zn‐Sn‐S precursor films under reactive atmosphere. Solar cells based on these CZTSSe absorbers have reached efficiencies up to 8% by using spin coating;^8^, ^9^ however, one of the drawbacks of spin coating is the low materials utilization because most of the ink dropped onto the substrate is spun away during spin coating. In this context, drop‐on‐demand inkjet printing is a promising approach allowing on‐demand patterning of materials with negligible materials waste; hence, significant reduction of raw materials cost can be achieved. For example, less than 20 μL ink is needed to build up a micrometer CZTSSe thin film absorber on an inch by inch substrate in this study. Furthermore inkjet printing can also be easily adapted to a roll‐to‐roll process, which is suitable for large‐scale production.^14^ For instance, the CZTSSe absorber reported here is printed on a large area (75 × 75 mm^2^) Mo coated substrate. Inkjet printing allows direct patterning without the requirements of any mask.^15^ Due to these advantages, lots of efforts have been focusing on using inkjet printing to fabricate organic solar cells and transistors.^15^, ^16^ However, there are only a few reports regarding the application of inkjet printing for CZTSSe and CIGSSe solar cells. A critical requirement for using inkjet printing is to develop a suitable ink in terms of viscosity and stability which leads to compact and homogeneous films. In 2011, Wang et al. reported the fabrication of a 5.04% efficient solar cell based on inkjet‐printed CIGSe thin film absorbers.^17^ Another use of inkjet printing related to CIGSe solar cells is reported by Hersh et al. who achieved 11.4% conversion efficiency with inkjet‐printed Ag contact grids compared to 14.8% conversion efficiency with standard evaporated Ni:Al contacts.^18^ We have recently shown that inkjet printing may also be feasible for depositing precursors for CZTSSe absorbers.^19^ It has been reported that sodium has a positive influence on the morphology as well as electronic properties of CZTSSe absorbers, thereby enhancing the solar cell performance.^9^, ^20^ In this work, we report on the development of CZTSSe absorbers with improved properties based on inkjet printing using a sodium containing Cu‐Zn‐Sn‐S precursor ink.

**Figure** **1**a shows an image of the Cu‐Zn‐Sn‐S precursor ink formulated by mixing metal salts and thiourea in DMSO. When loading the ink inside the print head, most of the nozzles work well as indicated by the drop view image displayed in Figure 1b. As it is well known, the wettability between the ink and the substrate plays a critical role for the formation of homogeneous films.^21^ Therefore, contact angle measurements were performed for both, the DMSO solvent and formulated Cu‐Zn‐Sn‐S ink on a Mo substrate. As a result, the contact angle was determined to be 21.6° for DMSO solvent as shown in Figure 1c, which is an indication of very good wetting behavior between DMSO and Mo. Figure 1d shows that an increase of the contact angle to 42.4° was observed for the Cu‐Zn‐Sn‐S precursor ink which is used for inkjet printing of CZTSSe absorbers in this work. The increase of contact angle is due to the enhancement of viscosity by adding metal salts to the DMSO solvent. The contact angle is still below 90°, suggesting the feasibility for the formation of a homogeneous film on Mo substrate by printing.

**Figure 1:**
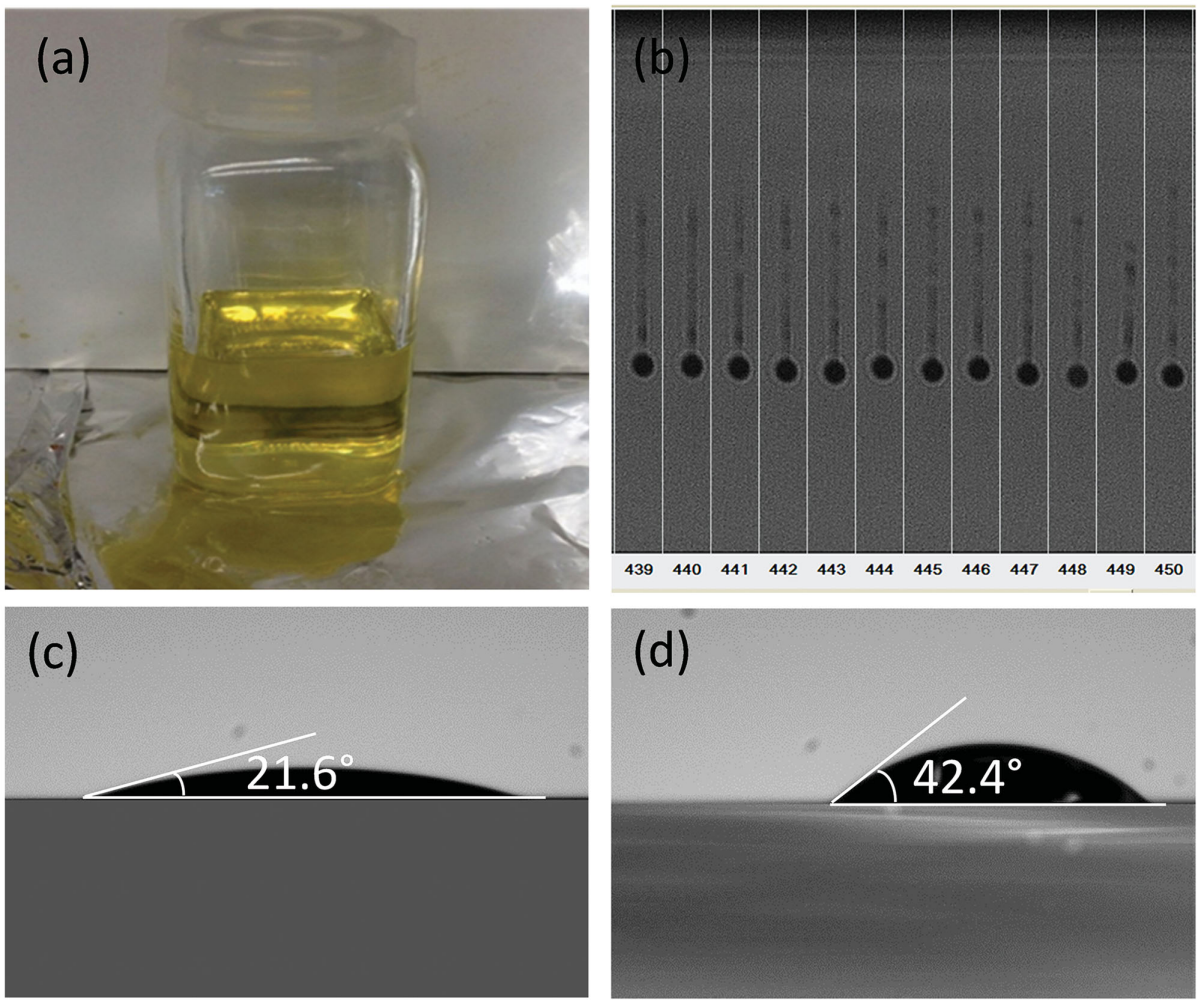
a) Photograph of Cu‐Zn‐Sn‐S precursor ink; b) drop view image of Cu‐Zn‐Sn‐S precursor ink in a KM520 print head; contact angle of c) DMSO and d) formulated Cu‐Zn‐Sn‐S precursor ink on Mo coated glass substrates.

Cu‐Zn‐Sn‐S layers were deposited on large area (75 × 75 mm^2^) Mo/glass substrates by inkjet printing followed by a 300 °C solvent removal step. CZTSSe absorbers layers were formed after annealing in presence of selenium. Grazing incident X‐ray diffraction (GIXRD) and Raman spectroscopy are used to characterize the structural properties of the thin films. **Figure** **2**a shows GIXRD patterns of inkjet‐printed Cu‐Zn‐Sn‐S layers after preheating and after annealing. Broad peaks are observed in the preheated Cu‐Zn‐Sn‐S sample. All peaks except for those corresponding to Mo substrate match quite well with kesterite CZTS in the preheated sample. However, it is still ambiguous whether all peaks originate from CZTS, Cu_2_SnS_3_, Cu_3_SnS_4_, or ZnS phases due to the overlapping of the X‐ray diffraction peaks of these compounds. In the GIXRD patterns of the annealed sample (shown in red in Figure 2a), the presence of characteristic peaks at around 36.2° and 43.1° which can be assigned to (211) and (213) lattice planes of kesterite CZTSSe indicates the formation of kesterite CZTSSe.^22^ However, the potential existence of Zn(S, Se) and Cu_2_Sn(S, Se)_3_ cannot be ruled out. The increasing GIXRD intensity and sharpening of the GIXRD peaks compared to the preheated Cu‐Zn‐Sn‐S sample suggest significant enhancement of the crystallinity due to annealing. Since the GIXRD pattern of the annealed sample was measured on the completed solar cells, peaks corresponding to window layer of ZnO were also detected. Since secondary phases like Cu*_x_*(S, Se) and SnS also show a major peak at around 31° and 31.6°, respectively, which is overlapping with the broad peak centered at around 31.8° labeled as CZTSSe (004/200), ZnO, and MoSe_2_, the existence of secondary phases of CuS and SnS cannot be ruled out. Furthermore, the ratio of S/[S + Se] is estimated to be around 0.09 based on the Vegard's Law using (112) peak.^23^

**Figure 2:**
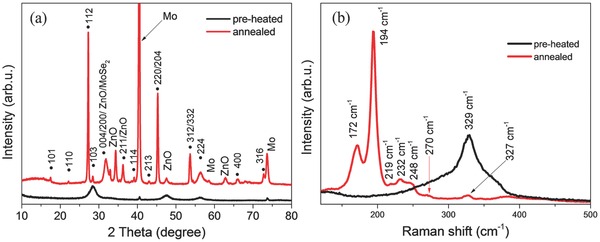
a) GIXRD patterns and b) Raman spectra of the preheated and annealed samples deposited from Cu‐Zn‐Sn‐S precursor inks; it should be noted that the GIXRD pattern and Raman spectrum of the annealed sample are obtained from the completed solar cell devices.

Figure 2b shows the Raman spectra of preheated Cu‐Zn‐Sn‐S and annealed samples. A broad peak centered at 329 cm^−1^ was observed in the as‐deposited Cu‐Zn‐Sn‐S sample, which can be assigned to CZTS.^24^, ^25^ The shift of the peak position from the most frequently reported range of 334–338 cm^−1^ could be related to the highly disordered Cu and Zn in the nonstoichiometric CZTS precursor film derived from the Cu‐Zn‐Sn‐S ink with Cu/(Zn + Sn) = 0.73 and Zn/Sn = 1.02.^24^ After annealing under selenium atmosphere, the main peak shifted to lower wave number of 194 cm^−1^ which can be assigned to the vibrational A symmetry mode of kesterite CZTSe.^26^, ^27^ On the other hand, the A mode of CZTS was also observed located at 327 cm^−1^. The observation of bimode behavior where both the A mode from CZTS and CZTSe are observed is common for CZTSSe.^6^, ^12^ Additional to the peaks corresponding to the A vibrational mode, the characteristic B (172 and 248 cm^−1^) and E (232 cm^−1^) modes of kesterite CZTSe were also detected. The peak at 270 cm^−1^ could be attributed either to the A mode of kesterite CZTS (284 cm^−1^ from theoretical calculation^26^ or the E mode of kesterite CZTS (281 cm^−1^ from the theoretical calculation.^26^ The shift of this peak from the calculated values could be explained by the partial substitution of S with Se in the CZTS, which is in agreement with the shift of the A mode observed at 327 cm^−1^ for CZTS. However, the existence of Cu(S_1−*x*_ Se*_x_*) phase that shows a peak between 263 and 278 cm^−1^ cannot be ruled out.^28^ Additionally, a minor peak located at 219 cm^−1^ was also detected, which could be attributed either to the E mode of kesterite or the *A*_g_ vibrational mode of a SnS secondary phase.^26^, ^29^

The morphologies of the preheated Cu‐Zn‐Sn‐S and annealed CZTSSe thin films were studied by scanning electron microscope (SEM). A compacted and crack‐free preheated Cu‐Zn‐Sn‐S thin film was observed (see Figure S1, Supporting Information). **Figure** **3**a shows a typical cross‐sectional SEM image of a CZTSSe solar cell. On top of the CZTSSe absorber are the CdS buffer layer, i‐ZnO and ZnO:Al window layers. The CZTSSe thin film absorber shows a layered structure with an ≈600–800 nm thick coarse grained layer on top and an around 400 nm thick fine grained layer underneath. The formation of layered structures is a common phenomenon when processing CZTSSe thin films via solution routes using organic solvents.^6^, ^8^, ^11^, ^12^, ^30^ A thick Mo(S,Se)_2_ interfacial layer (over 1 μm) was observed between the CZTSSe absorber layer and the Mo back contact. The formation of this Mo(S,Se)_2_ layer is due to the reaction between Mo and chalcogen diffusing through the CZTSSe layer during annealing or a direct reaction between CZTSSe itself and Mo.^31^ It is believed that a thin MoSe_2_ layer is helpful for the adhesion and formation of Ohmic contact in CIGSSe solar cells^32^ and the same may be true for CZTSSe cells.^32^ However, when the MoSe_2_ layer is too thick, the cell performance is lowered and adhesion may be poor.^27^ Energy‐dispersive X‐ray spectroscopy (EDX) line scanning profile reveals a near homogeneous elemental distribution across the large grain and fine nanoparticle layers. Moreover, the overall ratio of S/(S + Se) in the CZTSSe absorber is determined to be around 0.08 from the EDX profile, which is close agreement with the value of 0.09 as estimated from the XRD result.

**Figure 3:**
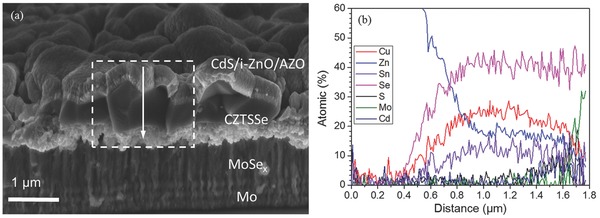
a) Cross‐sectional SEM image of a solar cell based on CZTSSe absorber and b) EDX line scanning profile across selected rectangular area.

Solar cells with Mo/CZTSSe/CdS/i‐ZnO/ZnO:Al/Ni:Al grids were fabricated based on the inkjet‐printed CZTSSe absorbers. **Figure** **4**a displays the current density–voltage (*J*–*V*) characteristics of the best solar cell under dark and standard AM1.5 sun simulator illumination. A power conversion efficiency of 6.4% was achieved on a total area of 0.5 cm^2^ with an open circuit voltage (*V*_OC_) of 431 mV, short current density (*J*_SC_) of 34.6 mA cm^−2^, and fill factor (FF) of 42.8%. The series resistance (*R*_S_d_), shunt resistance (*R*_Shunt_d_), and diode quality factor (*n*_d_) were estimated to be 5.7, 1230 Ω cm^2^, and 2.3, respectively, by fitting the dark *J*–*V* curve using the one diode model. Compared to the 11.1% efficient device reported by Todorov et al.^33^ where *V*_OC_ = 459.8 mV, *J*_SC_ = 34.5 mA cm^−2^, and FF = 69.8%, our device shows comparable *V*_OC_ and *J*_SC_ but much lower FF. The device performance is limited by the low FF which is due to the high series resistance under light (*R*_SL_) of the device. The *R*_SL_ is estimated to be 4.45 Ω cm^2^ under illumination, which is much higher than 0.40 Ω cm^2^ of the 11.1% efficient device.^33^ One of the main reasons for the high series resistance in our device is the thick MoSe_2_ layer (over 1 μm) in the back contact.^34^ Another reason for the high series resistance could be the fine grain layer in the CZTSSe absorber (see Figure 3a). To reduce or even avoid the fine grain layer in solution processed CZTSSe absorber, one of the solutions could be using water instead of organic as solvent.^35^ By considering a simple equivalent circuit consisting of a single diode, current source, and series resistance, it can be estimated that when the *R*_SL_ is reduced to 0.45 Ω cm^2^, the FF can be improved to 62.2% in our device. Accordingly, the power conversion efficiency of our device can be increased to 9.3%. Furthermore, among the eight cells on the same substrate, the efficiencies are in a range of 4.0%–6.4% with an average of 5.0% and standard deviation of 0.72%, indicating a reasonable homogeneity of the inkjet‐printed CZTSSe thin films.

**Figure 4:**
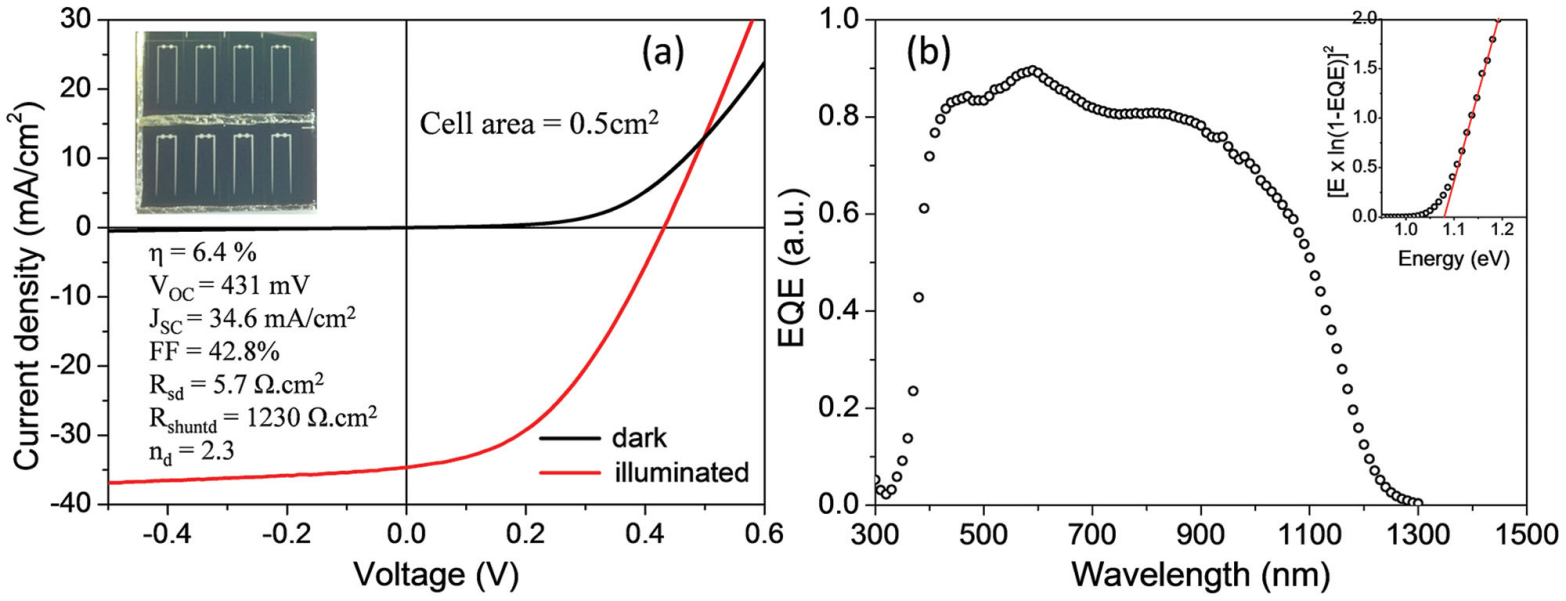
a) *J*–*V* characteristics of the best solar cell measured in dark and under illumination; device parameters are calculated based on the total area 0.5 cm^2^; Inset: photograph of eight devices on one substrate. b) EQE spectra of the best solar cell; the inset shows the bandgap estimated by extrapolating the linear part of the plot [*E* × ln (1 − EQE)]^2^ vs *E*.

Figure 4b depicts the external quantum efficiency (EQE) of the best device. The EQE reaches its maximum of 90% at the wavelength of around 600 nm and then gradually decrease towards the higher wavelength. The band gap of the absorber was estimated to be 1.08 eV by plotting the [*E* × ln(1 − EQE)]^2^ vs *E*, as shown in the inset of Figure 4b. The band gap of Cu_2_ZnSn(S*_x_* Se_(1−*x*)_)_4_ (CZTSSe) increases almost linearly with increasing S content.^36^ According to Bag et al.,^37^ the band of CZTSSe absorber with S/(S + Se) ≈ 0.03 is 1.04 eV. Therefore, the slightly higher band gap in our sample is in good agreement with the ratio of S/(S + Se) in our CZTSSe absorber.

In conclusion, we demonstrate that good quality CZTSSe thin film absorbers can be achieved by inkjet printing and reactive annealing. Solar cells with total area power conversion efficiencies up to 6.4% were achieved under AM1.52 without antireflection coating. The solar cell performance is limited by the low fill factor which is mainly due to the high series resistance of the device. It is expected that the solar cell performance could be further improved when reducing the series resistance by decreasing the thickness of the fine grained layer and the MoSe_2_ layer.

## Experimental Section

**Scheme 1:**
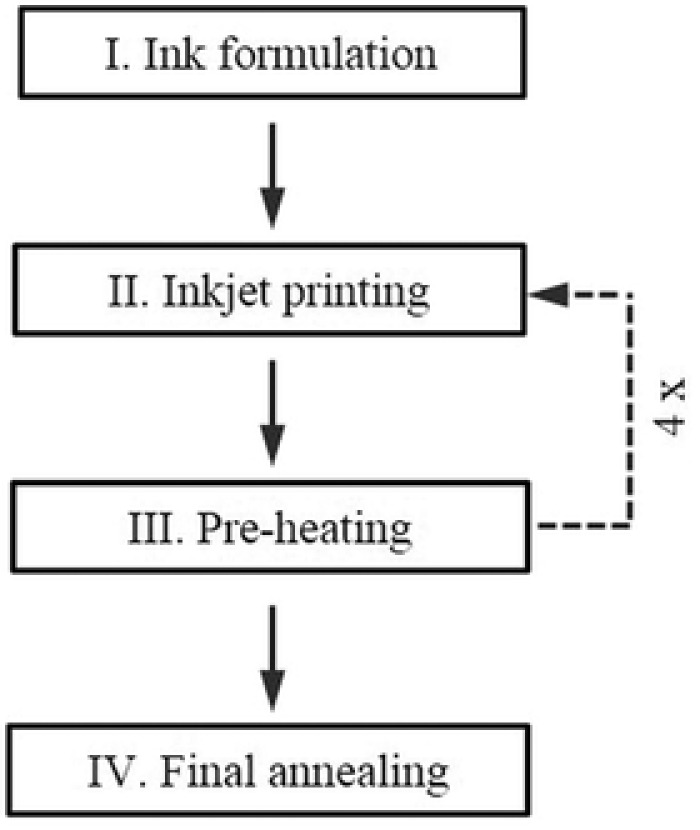
Formation procedures of CZTSSe thin films.

**Scheme** **1** shows the formation process of the CZTS(Se) thin films. First, the ink used for inkjet printing was formulated by mixing Copper chloride (Alfa Aesar, 98%), zinc acetate dihydrate (Sigma‐Aldrich, 98%), tin chloride dihydrate (Sigma‐Aldrich, 98%), thiourea (Merck, 99%), and sodium fluoride (0.14 m) were mixed in 12 mL DMSO with stirring overnight. The total amount of Cu, Zn, and Sn metal salts is 32.7 × 10^−3^ m. The metal ratios of the ink precursor were Cu/(Zn + Sn) = 0.73 and Zn/Sn = 1.02. Prior to printing, the wettability of the ink on Mo coated glass substrate was checked by measuring the contact angle. The ink was filtered by using an 800 nm polytetrafluoroethylene filter before loading into the ink container in the printer. The formulated ink was printed on a molybdenum (800 nm) coated glass substrate by using a PiXDRO LP50 printer from OTB Solar—Roth & Rau. The size of the substrate table is as large as an A4 paper, 210 mm in width, and 297 mm in length. Here, we used a 75 mm × 75 mm^2^ substrate. The print head is a Konica Minolta head with 512 piezoelectric nozzles, each capable of generating a nominal drop volume of 14 pL. It should be noted that the drop volume can be tuned by adjusting printing parameters such as the voltage applied to the print head and the viscosity of the ink. In our experiment, the applied on pulse voltage and off pulse voltage of the print head are 18 and 10 V, respectively; and the droplet volume is less than 20 pL. The printing speed was set to 4.8–7.2 m min^−1^. The resolutions in both the X and Y directions for the printing were 400 dpi. The substrate was sequentially cleaned under ultrasonication bath in acetone, ethanol, and water for 15 min, respectively. The as‐deposited C‐Z‐T‐S precursor thin films were baked on a preheated hot plate at 300 °C for 2 min after printing to remove the residual solvent. Based on the printing resolution and the droplet volume, the volume of ink needed for each printing on an inch by inch substrate is calculated to be less than 4 μL. To reach desired thickness, the inkjet printing and preheating steps were repeated four times. After that, precursor thin films were cut into 25 mm × 25 mm size and annealed under ambient pressure in a quartz tube furnace under selenium containing atmosphere at 560 °C for 20 min to allow the formation and crystal growth of CZTSSe thin film absorbers. A schematic diagram of the annealing furnace is shown in Figure S2 (Supporting Information). It should be noted that the quartz tube was evacuated and filled wih argon three times before starting heating. Argon was flowed through the quartz tube during the whole annealing process. Prior to the solar cells fabrication, the CZTSSe absorbers were soaked in 5 vol% HCl at 75 °C for 10 min to remove ZnS or ZnSe secondary phase^38^ and etched by 10% KCN for 3 min to remove Cu*_x_*(S,Se) phases. Solar cells were fabricated by chemical bath deposition of a CdS buffer layer, and by sputtering of i‐ZnO and aluminum doped ZnO window layers. A Ni/Al contact grid on top of the solar cell was deposited by evaporation using a shadow mask. Finally solar cells with an area of 0.5 cm^2^ were defined by mechanical scribing. It should be noted that no antireflection coating layer was applied.

*Characterization*: The contact angle was measured by using the “contact angle system OCA” from DataPhysics Instruments GmbH, Germany. The structure of the films was studied by GIXRD and Raman spectroscopy. GIXRD were operated in the 2*θ* range from 10° to 80° on a Bruker D8‐Advance X‐ray diffractometer with CuKα1 radiation at an incident angle of 5° using a step size of 0.02° and step time of 5 s. For the Raman measurements, a Dilor LabRam Raman setup was used. A He–Ne laser with a wavelength of 632.8 nm was used as an excitation source. To avoid laser heating the beam power was kept below 7 mW. Raman spectra were recorded in backscattering configuration with a microscope and a motorized XY stage. The micro‐Raman spectroscopy with a 50× objective was performed at room temperature. Silicon was used as a reference for the calibration. The morphologies of the layers were analyzed in a LEO 1530 GEMINI SEM of Zeiss. The SEM image was recorded at an acceleration voltage of 10 kV. Energy dispersive X‐ray spectroscopy analysis was performed in the LEO GEMINI 1530 field‐emission gun SEM with the operating voltage of 10 kV and a Thermo Noran X‐ray silicon drift detector (acquisition and evaluation software Noran System Seven). *I*–*V* characteristics were analyzed by using an in‐house class A sun simulator under standard test conditions (Air Mass (AM) 1.5G, 100 mW cm^−2^, and 23 °C). Quantum efficiency analysis has been performed using an illumination system including two sources (halogen and xenon lamps) and a Bentham TM300 monochromator (Bentham Instruments, Berkshire, UK). Reference measurements were performed on calibrated Si and Ge detectors.

## Supporting information

As a service to our authors and readers, this journal provides supporting information supplied by the authors. Such materials are peer reviewed and may be re‐organized for online delivery, but are not copy‐edited or typeset. Technical support issues arising from supporting information (other than missing files) should be addressed to the authors.

Supplementary
