# Supplementary material for: Inkjet‐Printed Cu2ZnSn(S, Se)4 Solar Cells
Source: Adv Sci (Weinh). 2015 May 5;2(6):1500028. doi: 10.1002/advs.201500028 (PMC5115396; doi:10.1002/advs.201500028)
Supplement: Supplementary file 1 — Supplementary [file ADVS-2-0j-s001.pdf]

Copyright WILEY-VCH Verlag GmbH & Co. KGaA, 69469 Weinheim, Germany, 2015.

ADVANCED SCIENCE
Open Access

Supporting Information

for *Adv. Sci.*, DOI: 10.1002/advs.201500028

Inkjet-Printed Cu_2 $ZnSn(S, Se)_4$ Solar Cells

Xianzhong Lin, Jaison Kavalakkatt, Martha Ch. Lux-Steiner, and Ahmed Ennaoui**

Supporting Information

Inkjet-printed $Cu_2ZnSn(S, Se)_4$ solar cells

Xianzhong Lin,[1] Jaison Kavalakkatt,[1] Martha Ch. Lux-Steiner[1,2] and Ahmed Ennaoui*[1]*

[1] Institute for Heterogeneous Material Systems
Helmholtz-Zentrum Berlin für Materialien und Energie
Hahn-Meitner-Platz 1, 14109 Berlin, Germany
[2] Freie Universität Berlin, Berlin, Germany

E-mail: lin.xianzhong@helmholtz-berlin.de; ennaoui@ helmholtz-berlin.de

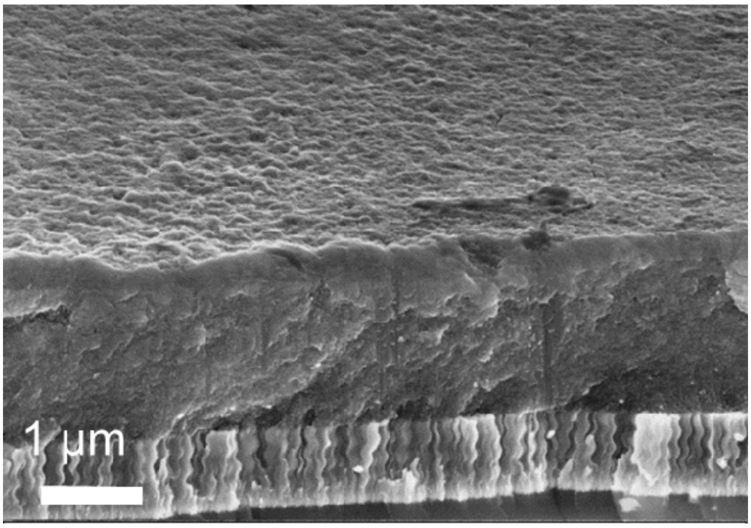

Figure S1 Cross-sectional scanning electron microscope image of pre-heated CZTS thin film with 8 times of printing.

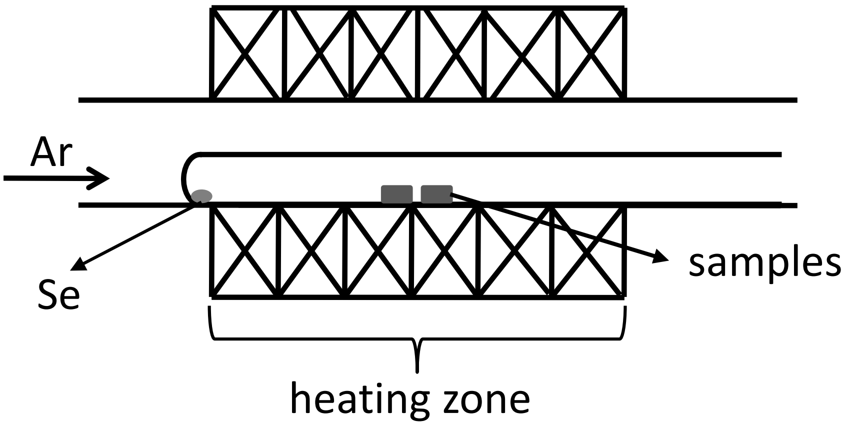

Figure S2 Quartz tube annealing furnace used for CZTSSe absorbers.

The annealing was done in a Gero split-tube furnace with one heating zone. The annealing temperature was controlled by a controlling device which allows programming the annealing profile as designed. The samples were loaded in a one end closed quartz tube which was placed inside a larger quartz tube, as shown in Figure S2. Selenium powder was placed at the closed end of the smaller quartz tube (see Figure S2). Both ends of the quartz tube in contact with the heating furnace were connected to gas-inlet and outlet, which allows the flowing of gas during annealing process. Before starting heating, the quartz tube was evacuated and filled with argon three times. The annealing was done under atmosphere pressure.
